# The Complete Chloroplast Genome Sequences of Six *Rehmannia* Species

**DOI:** 10.3390/genes8030103

**Published:** 2017-03-15

**Authors:** Shuyun Zeng, Tao Zhou, Kai Han, Yanci Yang, Jianhua Zhao, Zhan-Lin Liu

**Affiliations:** Key Laboratory of Resource Biology and Biotechnology in Western China (Ministry of Education), College of Life Science, Northwest University, Xi’an, 710069, China; zengsy.nwu@outlook.com (S.Z.); woody196@163.com (T.Z.); hank.nwu@outlook.com (K.H.); yycjyl@163.com (Y.Y.); yunjin1991@163.com (J.Z.)

**Keywords:** *Rehmannia*, chloroplast genome, repeat, positive selection, phylogeny

## Abstract

*Rehmannia* is a non-parasitic genus in Orobanchaceae including six species mainly distributed in central and north China. Its phylogenetic position and infrageneric relationships remain uncertain due to potential hybridization and polyploidization. In this study, we sequenced and compared the complete chloroplast genomes of six *Rehmannia* species using Illumina sequencing technology to elucidate the interspecific variations. *Rehmannia* plastomes exhibited typical quadripartite and circular structures with good synteny of gene order. The complete genomes ranged from 153,622 bp to 154,055 bp in length, including 133 genes encoding 88 proteins, 37 tRNAs, and 8 rRNAs. Three genes (*rpoA*, *rpoC2*, *accD*) have potentially experienced positive selection. Plastome size variation of *Rehmannia* was mainly ascribed to the expansion and contraction of the border regions between the inverted repeat (IR) region and the single-copy (SC) regions. Despite of the conserved structure in *Rehmannia* plastomes, sequence variations provide useful phylogenetic information. Phylogenetic trees of 23 Lamiales species reconstructed with the complete plastomes suggested that *Rehmannia* was monophyletic and sister to the clade of *Lindenbergia* and the parasitic taxa in Orobanchaceae. The interspecific relationships within *Rehmannia* were completely different with the previous studies. In future, population phylogenomic works based on plastomes are urgently needed to clarify the evolutionary history of *Rehmannia*.

## 1. Introduction

*Rehmannia* Libosch. ex Fisch. et Mey. is a small genus consisting of six species, among which five (*Rehmannia chingii*, *Rehmannia henryi*, *Rehmannia elata*, *Rehmannia piasezkii*, *Rehmannia solanifolia*) are endemic to China, while *Rehmannia glutinosa*, a famous and valuable species in Chinese traditional medicine, extends its distribution range from North China to Korea and Japan [1]. The systematic position of *Rehmannia* has been debated for years. It was traditionally placed in Scrophulariaceae based on morphological traits. Recently, molecular evidence indicated that Scrophulariaceae was polyphyletic [2]. *Rehmannia* was then transferred to Plantaginaceae [3] and later placed in Orobanchaceae as the second non-parasitic branch [4,5] or treated as an independent family [6]. Besides the uncertain familial placement of *Rehmannia*, interspecific relationships within the genus are also unsolved. Despite of the differences in some flower traits, the two tetraploid species *R. glutinosa* and *R. solanifolia* share identical chloroplast and nuclear haplotypes [7,8], inferring the possibility of the symnonym of one species. Similarly, evidence from morphology, pollen, allozyme, chemical composition, and molecular data support the theory that *R. piasezkii* and *R. elata* should also be considered one species [7,9,10]. Moreover, interspecific phylogenetic relationships are incongruent when constructed by different DNA fragments. Chloroplast fragments supported *R. chingii* was the basal taxon of the genus [5] while *R. piasezkii* was confirmed as the sister group to the remaining taxa within the genus by nuclear data [7,8].

The controversy in systematic position and interspecific relationships of *Rehmannia* partly lies in the lack of sufficiently effective data. Traditional morphological classification based on limited selected characters is often deeply affected by environmental and developmental factors of samples. For example, bracteoles absence or presence, considered as the critical trait to discriminate *R. piasezkii* from *R. chingii*, are not species-specific and variable among intraspecific individuals [9]. Although molecular data such as chloroplast and/or nuclear DNA fragments provide some information for the taxonomy of *Rehmannia* [5,7,8], phylogenetic analysis based on these limited data are usually unreliable for their low resolution. 

Most chloroplast (cp) genomes have a typical quadripartite structure with a pair of inverted repeats (IRs) separated by a large single-copy region (LSC) and a small single-copy region (SSC), and the genome size ranged from 120 to 160 Kb in length [11]. Previous studies indicated that the complete chloroplast genome sequences could improve the resolution at lower taxonomic level [12,13,14]. The Next Generation Sequencing (NGS) technique has enabled generating large amounts of sequence data at relatively low cost [15,16,17]. Up to now, approximately 644 plastid genomes in Viridiplantae have been sequenced and deposited in the National Center for Biotechnology Information (NCBI) Organelle Genome Resources. These massive data, together with the conservation of cp sequences, made it become a more increasingly used and effective tool for plant phylogenomic analysis than nuclear and mitochondrial genomes.

In this study, we sequenced, assembled, and characterized the plastomes of six *Rehmannia* species to verify the familial placement and evaluate the interspecific variation within the genus. These analyses will not only improve our understanding of the evolutionary mechanism of *Rehmannia* plastome and but also aid to clarify the ambiguous phylogenetic position of *Rehmannia.*

## 2. Materials and Methods

### 2.1. DNA Extraction and Sequencing

All samples used in the study were transplanted from their native habitats and cultivated in the greenhouse of Northwest University. No specific permits are required for sampling (Table 1). Healthy and fresh leaves from a single individual of the *Rehmannia* species were collected for DNA extraction.

The organelle-enriched DNAs of *R. glutinosa* were isolated using Percoll gradient centrifugation method [18] and CTAB extraction method. The DNA concentration was quantified using a NanoDrop spectrophotometer (Thermo Scientific, Carlsbad, CA, USA). -The DNA with concentration >30ng/μL was fragmented by mechanical interruption (ultrasonic), using PCR amplification to form a sequencing library. We sequenced the complete chloroplast genome of *R. glutinosa* with Illumina MiSeq platform at Sangon Biotech Co. (shanghai, China). A paired-end (PE) library with 265-bp insert size was constructed. Total genomic DNAs of other five *Rehmannia* species (*R. solanifolia*, *R. chingii*, *R. piasezkii*, *R. elata*, and *R. henryi*) were extracted with simplified CTAB protocol [19]. A paired-end (PE) library with 126-bp insert size was constructed using the Illumina PE DNA library kit and sequenced using an Illumina Hiseq 2500 by Biomarker technologies CO. (Beijing, China).

### 2.2. Chloroplast Genome Assembling and Annotation

Raw reads of *R. glutinosa* were trimmed to remove the potential low quality bases. Chloroplast genome was assembled using Velvet Assembler version 1.2.07 [20] and SPAdes [21]. Gaps and ambiguous (N) bases of the plastome were corrected using SSPACE premium version 2.2 [22]. The annotation of the plastome was performed with online tool CpGAVAS (http://www.herbalgenomics.org/cpgavas) and the gene homologies were confirmed by comparing with the NCBI’s non-redundant (Nr) protein database, Cluster of Orthologous Group (COG), CDD (https://www.ncbi.nlm.nih.gov/Structure/cdd/cdd.shtml), PFAM (http://xfam.org), SWISS-PROT (http://web.expasy.org/docs/swiss-prot_guideline.html), and TREMBL (http://www.bioinfo.pte.hu/more/TrEMBL.htm) databases. The raw reads of five other *Rehmannia* species were quality-trimmed using CLC Genomics Workbench v7.5 (CLC bio, Aarhus, Denmark) with default parameters. Reference-guided assembly was then performed to reconstruct the chloroplast genomes with the program MITObim v1.7 [23] using *R. glutinosa* as the reference. The cpDNA annotation was conducted using the program GENEIOUS R8 (Biomatters Ltd., Auckland, New Zealand), and used the plastome of *R. glutinosa* as the reference, coupled with manual adjustment for start/stop codons and for intron/exon borders. Transfer RNAs (tRNAs), ribosomal RNAs (rRNAs), and coding sequences were further confirmed, and in some cases, manually adjusted after BLAST searches. The circle maps of six *Rehmannia* plastomes were obtained using Organellar Genome DRAW software (OGDRAW, http://ogdraw.mpimp-golm.mpg.de) [24]. Ambiguous (N) bases and large insertion/deletion fragment (*rpoC2*) were validated by PCR amplification and Sanger sequencing (Table S1).

### 2.3. Sequence Analysis and Repeat Structure

Multiple alignments of six *Rehmannia* plastomes were carried out using MAFFT version 7.017 [25]. Full alignments with annotation were visualized using the mVISTA software [26]. Genetic divergence parameter (*p*-distance) was calculated by MEGA 6.0 [27]. The percentage of variable characters for each noncoding region with an aligned length >200 bp in the genome was calculated as described in Zhang et al. [28]. Dispersed, tandem and palindromic repeats were determined by the program REPuter [29] (http://bibiserv.techfak.uni-bielefeld.de/reputer/manual.html) with a minimal size of 30 bp and >90% identity (Hamming distance equal to 3) between the two repeats. Gap size between palindromic repeats was restricted to a maximal length of 3 kb. Overlapping repeats were merged into one repeat motif whenever possible. Tandem Repeats Finder [30] (http://tandem.bu.edu/trf/trf.html) was used to identify tandem repeats in the six *Rehmannia* plastomes with default settings. A given region in the genome was designated as only one repeat type, and tandem repeat was prior to dispersed repeat if one repeat motif could be identified as both tandem and dispersed repeats.

### 2.4. Selective Pressure Analysis

Signals of natural selection were evaluated for all chloroplast genes located outside of IR region. Selective pressures, nonsynonymous to synonymous ratios (Ka/Ks), were computed with codeml tool of PAML package [31].

### 2.5. Comparative Genome Analysis

The whole plastomes of *Rehmannia* and 17 representatives of Lamiales species, including six Lamiaceae species, five Orobanchaceae species, and five species from other families (Table 2), were aligned separately by using MAUVE as implemented in Geneious with default settings [32] to test and visualize the presence of genome rearrangements and inversions

### 2.6. Phylogenomic Analyses

The chloroplast genome sequences of six *Rehmannia* species, together with 17 Lamiales species (Table 2), were aligned with the program MAFFT version 7.017 [25] and adjusted manually when necessary. In order to test the utility of different cp regions, phylogenetic analyses were performed based on the following four datasets: (1) the complete cp DNA sequences, (2) a set of the common protein coding genes (PCGs), (3) the large single copy region, and (4) the small single copy region. Maximum likelihood (ML) analyses were implemented in RAxML version 7.2.6 [33]. RAxML searches relied on the general time reversible (GTR) model of nucleotide substitution with the gamma model of rate heterogeneity. Non-parametric bootstrapping test was implemented in the ‘‘fast bootstrap’’ algorithm of RAxML with 1000 replicates. Bayesian analyses were performed using the program MrBayes version 3.1.2 [34]. The best-fitting models were determined by the Akaike Information Criterion [35] as implemented in the program Modeltest 3.7 [36]. The Markov chain Monte Carlo (MCMC) algorithm was run for 200,000 generations with trees sampled every 10 generations for each data partition. The first 25% of trees from all runs were discarded as burn-in, and the remaining trees were used to construct majority-rule consensus tree. In all analyses, *Olea europaea* was set as an outgroup.

## 3. Results

### 3.1. Genome Sequencing, Assembly, and Validation

Illumina paired-end sequencing generated 1 Gb raw reads for *R. glutinosa*, accounting for 91.1% of the total reads with average length of 265 bp. The sequencing depth and coverage were approximately 6600 and 1583.5, respectively. Using the Illumina Hiseq 2500 system (Biomarker technologies CO.), five other *Rehmannia* species produced large data for each species from 25,724,095 (*R. chingii*) to 31,171,142 (*R. henryi*) clean reads (126 bp in average reads length). All paired-end reads were mapped to the reference plastome of *R. glutinosa* with the mean coverage of 119.6× to 141.8× (Table 1). Gaps were validated by using PCR-based sequencing with seven pairs of primers (Table S1). All six *Rehmannia* plastome sequences were deposited in GenBank (accession numbers: KX426347, KX636157- KX636161) (Table 1, Table 2).

### 3.2. Complete Chloroplast Genomes of *Rehmannia* Species

**Figure 1:**
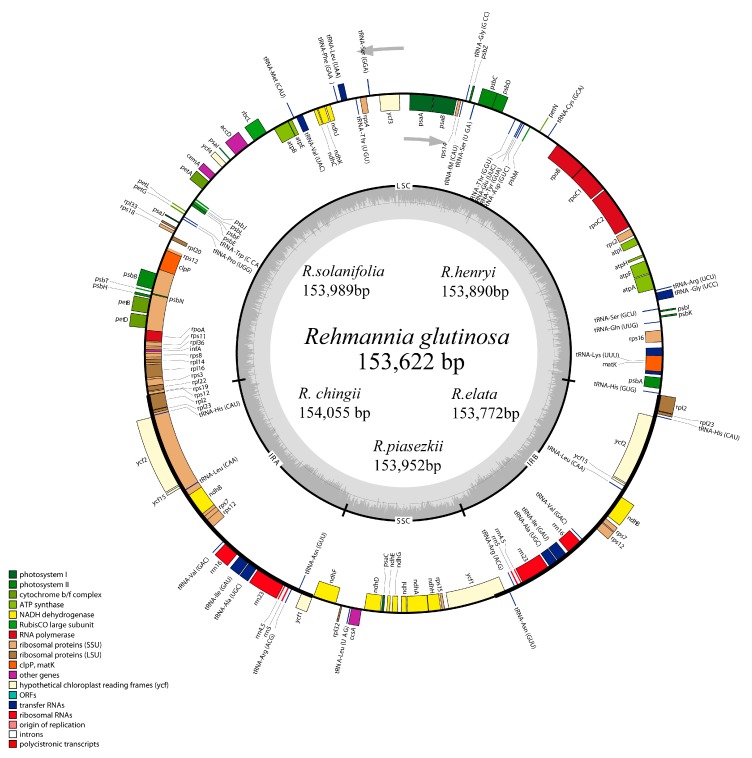
Gene map of *Rehmannia* chloroplast genomes. Genes shown outside the outer circle are transcribed clockwise and those inside are transcribed counterclockwise. Genes belonging to different functional groups are color coded. Dashed area in the inner circle indicates the GC content of the chloroplast genome. ORF: open reading frame.

The six chloroplast genomes of *Rehmannia* ranged in size from 153,622 bp (*R. glutinosa*) to 154,055 bp (*R. chingii*). All of them exhibited a typical quadripartite structure consisting of a pair of IRs (25,666–25,735 bp) separated by the LSC (84,605–84,966 bp) and SSC (17,579–17,680 bp) regions (Figure 1). These six plastomes are highly conserved in gene content, gene order, and intron number. The overall GC content was about 38.0%, almost identical with each other among *Rehmannia* species (Table 2). The *Rehmannia* plastomes contained 133 genes, of which 115 occurred as a single copy and 18 were duplicated in the IR regions (Table 3). The predicted functional genes of each species were comprised of 88 protein-coding genes, 37 tRNA genes, and eight rRNA genes (Table 2). Sixteen genes (*rpl2*, *ndhB*, *petD*, *petB*, *ndhA*, *ndhB, rpl16*, *rpoC1*, *atpF1*, *rps16*, *trnA*-*UGC*, *trnG*-*GCC*, *trnI*-*GAU*, *trnK*-*UUU*, *trnL*-*UAA*, and *trnV*-*UAC*) had one intron, while three genes (*rps12*, *clpP*, and *ycf3*) contained two introns (Table 3). The *rps12* gene was a unique gene with 3′ end exon and intron located in the IR region, and the 5′ end exon in the LSC region. Unusual initiator codons were observed in *ndhD* with ATC and *rps19* with GTG in *Rehmannia* plastomes. Overlaps of adjacent genes were found in the complete genome, for example, *rps3*-*rpl22*, *atpB*-*atpE*, and *psbD*-*psbC* had a 16 bp, 4 bp, and 53 bp overlapping region, respectively. Large indels were detected in the *rpoC2* gene, which caused the gene size to vary from 2916 bp to 4185 bp among the six species (Figure S1).

### 3.3. IR Boundary Changes and Gene Rearrangement

**Figure 2:**
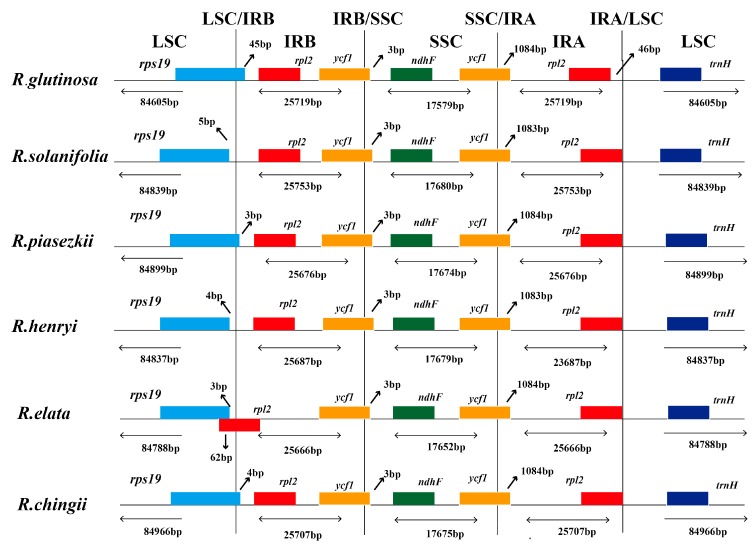
Comparison of the borders of large single-copy (LSC), small single-copy (SSC), and inverted repeat (IR) regions among the chloroplast genomes of six *Rehmannia* species. The location of two parts of inverted repeat region (IRA and IRB) was referred to Figure 1.

The IR region of six *Rehmannia* chloroplast genomes was highly conserved, but structure variation was still found in the IR/SC boundary regions. To elucidate the potential contraction and expansion of IR regions, we compared the gene variation at the IR/SSC and IR/LSC boundary regions of the six plastomes. The genes *rps19*-*rp12*-*trnH* and *ycf1*-*ndhF* were located in the junctions of LSC/IR and SSC/IR regions. Two copies of the *ycf1* gene crossed SSC/IRa and SSC/IRb with 3 bp in the SSC region and 1083/1084 bp in the IRa region, respectively (Figure 2). Compared to the relatively fixed location of the *ycf1* and *trnH* gene in all chloroplast genomes, the LSC/IR boundary regions were more variable. The rps19 gene in *R. glutinosa*, *R. piasezkii*, and *R. chingii* crossed the LSC/IRb region with 45 bp, 3 bp, and 4 bp located at the IRb region while the intergenic spacer of *rps19*-*rps12* extended 3 bp, 4 bp, or 5 bp to the LSC region in *R. elata*, *R. henryi*, and *R. solanifolia*, respectively. The *rpl2* gene of *R. elata*, commonly located in the IRb region in *Rehmannia*, extended 65 bp into the LSC region and overlapped with the rps19 gene by 62 bp. To identify the potential genome rearrangements and inversions, the chloroplast genome sequences of six *Rehmannia* species, *Arabidopsis thaliana* and 16 core Lamiales taxa, were selected for synteny analyses (Table 2). No gene rearrangement and inversion events were detected in *Rehmannia*, except *Cistanche deserticola* with structure variation of a 4 kb fragment. (Supplementary Figure S2).

### 3.4. Repetitive Sequences

**Figure 3:**
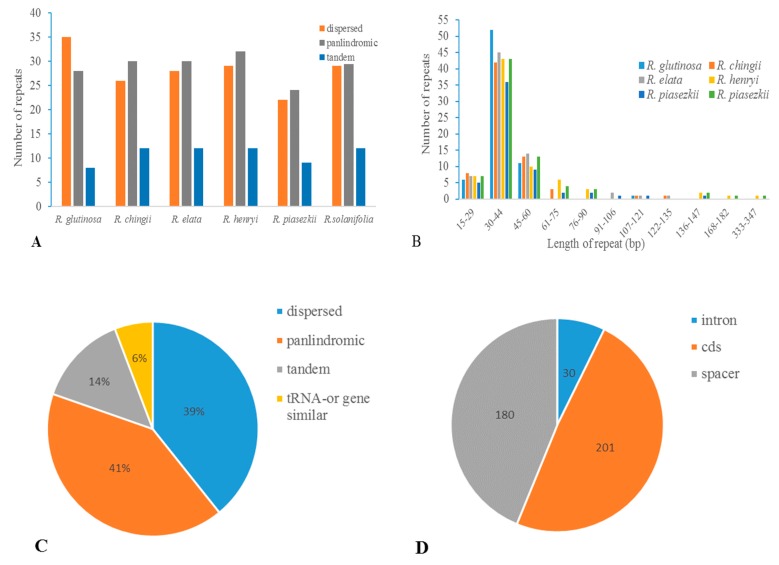
Analyses of repeated sequences in *Rehmannia* plastomes. (**A**) Number of three repeat types in the six chloroplast genomes; (**B**) Frequency of repeat sequences by length; (**C**) Frequency of repeat types; (**D**) Location of the all repeats from six species.

We classified sequence repeat motifs into three categories: dispersed, tandem, and palindromic repeats. For all repeat types, the minimal cut-off identity between two copies was set to 90%. The minimal repeat size investigated were 30 bp for dispersed, 15 bp for tandem and 20 bp for palindromic repeats, respectively. In total, 411 repeats were detected in *Rehmannia* plastomes (see Supplementary Table S2, Figure 3). Among these repeats, 24 were verified to be associated with two copies of tRNA (e.g., *trnG-UCC*) or gene duplication (e.g., *psaA*/*psaB*) and subsequently considered as tRNA or gene similarity repeats [37] due to their similarity in gene functions. Numbers of the three repeat types were similar among these six plastomes (Figure 3A) and their overall distribution in the plastome was highly conserved. Generally, palindromic repeats were the most common, while tandem repeats were the least in *Rehmannia* except *R. glutinosa* with dispersal repeats as the most common. The majority of repeats ranged in size from 30 bp to 44 bp (Figure 3B), even though the defined smallest size is 15 bp and 30 bp for tandem and dispersed repeats, respectively. The longest repeat is a palindromic repeat of 341 bp in *R. henryi* and *R. solanifolia*. Dispersed repeats had a wider size range (from 30 to 126 bp) than other repeat types. Palindromic repeat, accounting for 41% of total repeats, was the most common, followed by dispersed (39%), tandem (14%), and tRNA or gene similarity (6%) types (Figure 3C). A minority of repeats was found in intron (7.3%), while the majority were located in coding regions (48.9%) (such as gene *ycf2*, *rps18*, *rps11* and *rpoC*2) and intergenic spacers (43.8%) (Figure 3D). 

### 3.5. Sequence Divergence and Divergence Hotspot

**Figure 4:**
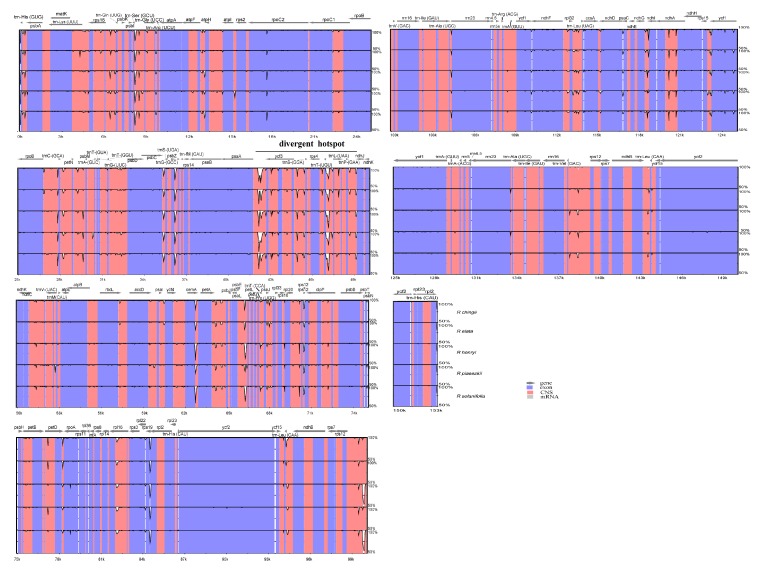
Visualization of alignment of the six *Rehmannia* species chloroplast genome sequences. VISTA-based identity plots showed sequence identity of six sequenced chloroplast genomes with *R. glutinosa* as a reference. The sequence similarity of the aligned regions is shown as horizontal bars indicating the average percent identity between 50% and 100% (shown on the y-axis of the graph). The x-axis represents the coordinate in the chloroplast genome. The divergent hotspot region is indicated in the chloroplast genome. Genome regions are color coded as protein coding, rRNA coding, tRNA coding or conserved noncoding sequences (CNS).

**Figure 5:**
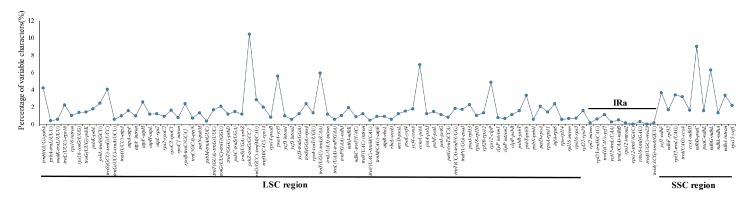
Percentage of variable characters in aligned noncoding regions of the six plastid genomes.

To elucidate the level of the genome divergence, sequence identity among *Rehmannia* cpDNAs were plotted using the program mVISTA with *R. glutinosa* as a reference. The whole aligned sequences showed high similarities with only a few regions below 90%, suggesting that *Rehmannia* plastomes were rather conserved (Figure 4). As expected, the IRs regions were more conserved than the single-copy regions, and the coding regions were less divergent than the non-coding regions. One divergent hotspot region in LSC (*psbA*-*ndhJ)* region was identified (Figure 4). The complete plastome sequence divergence of six species, estimated by *p*-distance, ranged from 0.002 to 0.004 with the average value of 0.0028. We also compared the sequence divergence among the different noncoding regions. Among the 98 noncoding regions, the percentage of variation ranged from 0 to 10.41% with an average of 1.7. Nine noncoding regions had over 4% variability proportions, such as *trnH(GUG)*-*psbA*, *trnS(GCU)*-*trnG(UCC)*, *psbZ*-*trnG(GCC)*, *psaA*-*ycf3*, *trnT(UGU*)-*trnL(UAA)*, *cemA*-*petA*, *rps12*-*clpP*, *nhdD*-*psaC*, and *ndhG*-*ndhL* (Figure 5). These divergence hotspot regions provided abundant information for marker development in phylogenetic analyses of *Rehmannia* species.

### 3.6. Selective Pressure Analysis

To estimate selection pressures among *Rehmannia* species, ratios of nonsynonymous (Ka) versus synonymous (Ks) substitutions were calculated for 79 protein-coding genes, generating 241 pairwise valid combinations (Table S3). The Ka/Ks ratios of the remaining comparisons were not available for Ks = 0. Three genes (*accD*, *rpoA*, *rpoC2*) located in the LSC region had Ka/Ks ratios above 1.0, which might indicate positive selection (Table S3).

### 3.7. Phylogenomic Analysis

**Figure 6:**
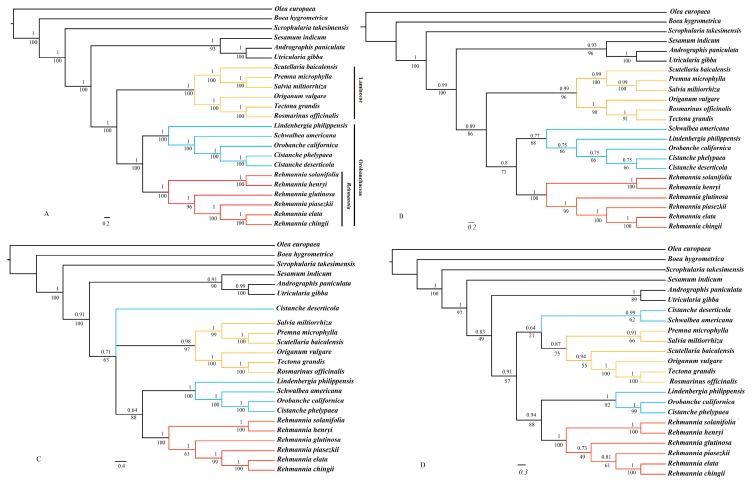
Phylogenetic trees of 23 species as determined from different data partitions. Support values are shown for nodes as Bayesian inference posterior probability (above branches)/maximum likelihood bootstrap (below branches). Branch lengths were calculated through Bayesian analysis, and scale bar denotes substitutions per site. (**A**) the whole chloroplast genomes; (**B**) Protein coding genes; (**C**) LSC region; (**D**) SSC region. Red represents *Rehmannia* species; Blue represents other species of Orobanchaceae; Orange represents Lamiaceae species.

To identify the phylogenetic position of *Rehmannia* within the Lamiales, four datasets (PCGs, the LSC region, the SSC region, and the whole plastome with one IR region removed) from the six *Rehmannia* plastomes and 17 published plastomes were used to reconstruct phylogenetic relationships with *O. europaea* as an outgroup (Table 2). These 22 ingroups represented seven core families in Lamiales [36]. The phylogenetic tree based on the same dataset using ML and Bayesian method had the identical topological structure with possibly different support values (Figure 6). There were no obvious conflicts between phylogenetic trees built by different partitions of the plastomes. Familial relationships based on the complete plastomes were quite identical to those with rapid evolving cp fragments as previously reported [38]. Along with the increase of sequence length, resolution power of main branches was dramatically improved. Each lineage of the phylogenetic tree with the whole plastome was well-supported with 100% bootstrap value or the Bayesian posterior probability of one (Figure 6A). Phylogenetic status of the parasitic species in Orobanchaceae was contradictive in trees based on PCGs and the LSC/SSC region (Figure 6B–D). But when all plastomes were used, parasitic species formed a monophyletic group sister to *Lindenbergia*, the non-parasitic species in Orobanchaceae (Figure 6). In all phylogenetic trees, six *Rehmannia* species were clustered into one monophyletic group sister to other Orobanchaceae taxa with high support value (Figure 6). Trees based on PCGs and the whole plastomes indicated that the Orobanchaceae clade including *Rehmannia* was sister to the Lamiaceae group. In terms of interspecific relationships of *Rehmannia*, four phylogenetic trees based on different datasets consistently showed the same topology with moderate to high support values: *R. solanifolia* and *R. henryi* were grouped into one branch sister to the remain species. *R. glutinosa* and *R. piasezkii* were successive sis*ters to R. elata-R. chingii* clade (Figure 6).

## 4. Discussion

### 4.1. Genome Characteristics and Sequence Differences

Here we determined the complete plastid genome sequences from six *Rehmannia* species using Illumina sequencing technology. Although plastomes were highly conserved in terms of genomic structure and size, the IR/SC junction position variation was observed in *Rehmannia.* This may be caused by the contraction or expansion of the IR region, a common evolutionary phenomenon in plants [39,40,41]. As for gene contents, the same set of 88 protein-coding genes were shared by six species of *Rehmannia* species and highly conserved in aspect of gene number, gene function, gene order, and GC-content. Noncanonical start codons observed in this study could be also found in other angiosperms [42] and tree fern plants [43].

The presence of repeats in plastomes, especially in intergenic spacer regions, has been reported in all published angiosperm lineages. Compared with other angiosperm species, the number of repeats in *Rehmannia* is rather high. In all, more than 400 repeats were detected in *Rehmannia* plastomes. Previous researches have suggested that repeat sequences may play roles in rearranging sequences and producing variation through illegitimate recombination and slipped-strand mispairing [37,44,45]. However, we detected no structural rearrangements or gene loss-and-gain events in *Rehmannia* plastomes. But the regions with high divergence were generally rich in repeat units. For example, variable types of repeats could be found within the noncoding region of *psbZ*-*trnG* (GCC), *psaA*-*ycf3* and *trnS(GCU)*-*trnG(UCC)* gene. Lamiales has the highest diversification rates among angiosperms [46]. Thus the high repeat number in *Rehmannia* might be ascribed to the increased evolutionary rates. 

Sequences of *Rehmannia* plastomes were conserved in most of regions with sequence identities above 90%. As expected, the noncoding regions exhibited higher divergent levels than the coding regions, and the single copy regions had higher variation than the IR regions. The *rpoC2* gene is an exception with lower sequence identity due to various indels, as also reported in grasses [47]. One divergent hotspot region associated with a tRNA cluster in LSC (*trnS(GGA)*-*trnP(GAA)*) region was identified, which is inconsonant to other herb plastomes [47,48]. We also found nine highly variable noncoding regions with variation percentages above 2%. Previous studies have also shown that noncoding regions of plastomes could be successfully used for phylogenetic studies in angiosperms [49,50]. Additional work is still necessarily needed to verify whether these highly variable regions could potentially be used as molecular targets for future phylogenetic and/or population genetics studies of *Rehmannia* species.

Our analysis indicated that three genes (*accD*, *rpoA* and *rpoC2*) were under positive selection, of which, *rpoA* and *rpoC2* were reported in Annonaceae [51]. The *accD* gene encodes a plastid-coded subunit of heteromeric acetyl-CoA carboxylase (ACCase), a key enzyme involved in fatty acid biosynthesis in plants [52]. The genes *rpoA* and *rpoC2* encode *α* and *β”* subunit of plastid-encoded plastid RNA polymerase (PEP), respectively, a key protein responsible for most photosynthetic gene expression [53]. Field works and photosynthetic physiological study suggested that the six species of *Rehmannia* might have divergent habitats and adapt to different light intensity (unpublished data). These genes experienced natural selection might play an important role in the evolution and divergence of *Rehmannia*.

### 4.2. Phylogenetic Implications

*Rehmannia* was traditionally placed in Scrophylariaceae s.l. In our study, phylogenetic trees based on four plastome datasets generated similar topological structures except for the SSC dataset, possibly due to fewer informative sites than others. All *Rehmannia* species were clustered into a monophyletic group with high support value (BI = 1, BS = 100%), and sister to the clade of the parasitic Orobanchaceae species and *Lindenbergia philippehsis* rather than a genus of Scrophulariaceae. Chloroplast fragments analyses also suggested that *Rehmannia*, together with *Triaenophora*, represented the branch sister to Orobanchaceae s.l (including *Lindenbergia*), or a new familial clade [5]. The phylogenetic placement of *Rehmannia*-Orobanchaceae clade in Lamiales remains uncertain because of the contradictive results of this clade and its related taxa of Paulowniaceae and Phrymaceae from cp fragments data [5,38]. For the lack of plastome information of Paulowniaceae and Phrymaceae species, the status of *Rehmannia*-Orobanchaceae is still unresolved in our study. But familial relationships of Lamiales taxa in this study were definite and identical to those verified by the rapid evolving cp gene fragments [38]. Therefore, analyses of the entire plastomes significantly contribute to species identification and phylogenetic studies of angiosperms [14,54,55].

Recent studies using chloroplast or nuclear gene fragments indicated that two pairs of species groups, *R. ela*ta-*R. piasezkii*, *R. glutinosa*-*R. solanifolia*, had completely identical sequences, respectively and could be treated as two species [7,9,10]. *R. chingii* [5] or *R. piasezkii* [7,8] were considered as the basal taxon of the genus. But our phylogenetic analyses of *Rehmannia* plastomes were inconsistent with any of these works, inferring all *Rehmannia* taxa as separate species (Figure 6). This might be ascribed to the insufficient informative characters of cp/nuclear gene fragments generating the phylogenetic trees with low support values. Of course, the entire plastomes with massive characters may also result in strong systematic biases for limited sampling [56,57,58]. In future, we plan to analyze plastomes at population level to elucidate the phylogenetic relationships of *Rehmannia* species. These comparative genomic analyses will not only provide insights into the chloroplast genome evolution of *Rehmannia*, but also offer valuable genetic markers for population phylogenomic study of *Rehmannia* and its close lineages.

## Figures and Tables

**Table 1:** Sample information of six *Rehmannia* species in this study.

Species	Location	Longitude	Latitude	Clean reads	Mean coverage
*R. glutinosa*	Yulin, Shaanxi, China	110.57	37.77	3,721,846	1583.5×
*R. henryi*	Yichang, Hubei, China	10.68	31.31	31,171,142	119.6×
*R. elata*	Amsterdam, Holland	4.88	52.36	26,976,944	137.1×
*R. piasezkii*	Shiquan, Shaanxi, China	108.63	32.04	26,815,865	125.3×
*R. chingii*	Lishui, Zhejiang, China	120.15	28.64	25,724,095	131.5×
*R. solanifolia*	Chengkou, Chongqing, China	108.62	1.54	29,076,484	141.8×

**Table 2:** Summary of chloroplast (cp) genomic data of all Lamiales taxa used in the study. The numbers in parenthesis indicate the genes duplicated in the inverted repeat (IR) regions.

Taxon	Species	GenBank	Length	LSC	SSC	IR	Gene	PCG	tRNA	rRNA	GC (%)
Orobanchaceae	*Rehmannia glutinosa* (Gaetn.) Libosch. ex Fisch. et Mey.	KX636157	153622	84605	17579	25719	133	88	37 (7)	8 (4)	38
	*Rehmannia chingii* Li.	KX426347	154055	84966	17675	25707	133	88	37 (7)	8 (4)	38
	*Rehmannia henryi* N.E. Brown	KX636158	153890	84837	17679	25687	133	88	37 (7)	8 (4)	37.9
	*Rehmannia elata* N.E. Brown	KX636161	153772	84788	17652	25666	133	88	37 (7)	8 (4)	38
	*Rehmannia piasezkii* Maxim.	KX636160	153952	84899	17674	25676	133	88	37 (7)	8 (4)	37.9
	*Rehmannia solanifolia* Tsoong et Chin	KX636159	153989	84839	17680	25735	133	88	37 (7)	8 (4)	37.9
	*Cistanche phelypaea* (L.) Coutinho	NC_025642	94380	32648	8646	26543	99	30	42 (9)	8 (4)	36.6
	*Cistanche deserticola* Ma	KC_128846	102657	49130	8819	22354	106	31	36 (7)	8 (4)	36.8
	*Orobanche californica* Cham. & Schltdl.	NC_025651	120840	62000	8516	25162	123	45	41 (6)	8 (4)	36.7
	*Lindenbergia philippensis* (Cham.) Benth.	NC_022859	155103	85594	17885	25812	137	85	37 (7)	8 (4)	37.8
	*Schwalbea americana* L.	HG_738866	160910	84756	18899	28627	128	82	37 (7)	8 (4)	38.1
Lamiaceae	*Rosmarinus officinalis* L.	KR_232566	152462	83355	17969	25569	134	86	37 (7)	8 (4)	38
	*Salvia miltiorrhiza* Bge.	NC_020098	153953	85318	17741	25447	134	86	37 (7)	8 (4)	37.9
	*Origanum vulgare* L.	JX_880022	151935	83135	17727	25533	134	86	37 (7)	8 (4)	37.8
	*Tectona grandis* L.F.	NC_020431	151328	82695	17555	25539	133	87	37 (7)	8 (4)	38
	*Premna microphylla*Turcz	NC_026291	155293	86078	17689	25763	133	87	37 (7)	8 (4)	37.9
	*Scutellaria baicalensis* Georgi	KR_233163	152731	83946	17477	25654	132	87	36 (7)	8 (4)	38.4
Scrophulariaceae	*Scrophularia takesimensis* Nakai	KM_590983	152425	85531	17938	23478	132	88	36 (6)	8 (4)	38.1
Gesneriaceae	*Boea hygrometrica* (Bunge) R. Br.	NC_016468	153493	84698	17903	25446	145	85	36 (7)	8 (4)	37.6
Acanthaceae	*Andrographis paniculata* (Burm. f.) Nees	NC_022451	150249	82459	17110	25340	132	87	37 (7)	8 (4)	38.3
Lentibulariaceae	*Utricularia gibba* L.	NC_021449	152113	81818	14187	27904	133	87	37 (6)	8 (4)	37.6
Pedaliaceae	*Sesamum indicum* Linn.	JN_637766	153324	85170	17872	25141	134	87	37 (7)	8 (4)	38.2
Oleaceae	*Olea europaea* L.	GU_931818	155889	86614	17791	25742	133	87	37 (7)	8 (4)	37.8

LSC: large single-copy; SSC: small single-copy; PCG: protein coding genes; tRNA: transfer RNA; rRNA: ribosomal RNA

**Table 3:** Gene list of plastomes of six *Rehmannia* species.

Category	Group	Name
Photosynthesis related genes	Rubisco	*rbcL*
	Photosystem I	*psaA*, *psaB*, *psaC*, *psaI*, *psaJ*
	Assembly/stability of photosystem I	** *ycf3*
	Photosystem II	*psbA*, *psbB*, *psbC*, *psbD*, *psbE*, *psbF*, *psbH*, *psbI*, *psbJ*, *psbK*, *psbL*, *psbM*, *psbN*, *psbT*, *psbZ*
	ATP synthase	*atpA*, *atpB*, *atpE*, * *atpF*, *atpH*, *atpI*
	cytochrome b/f complex	*petA*, * petB, * petD, *petG*, *petL*, *petN*
	cytochrome c synthesis	*ccsA*
	NADPH dehydrogenase	* ndhA, *^,a^ *ndhB*, *ndhC*, *ndhD*, *ndhE*, *ndhF*, *ndhG*, *ndhH*, *ndhI*, *ndhJ*, *ndhK*
Transcription and translation related genes	transcription	*rpoA*, *rpoB*, *rpoC1, *rpoC2*
	ribosomal proteins	*rps2*, *rps3*, *rps4*, ^a^ *rps7*, *rps8*, *rps11*, ** *rps12*, *rps14*, *rps15*, *rps16, *rps18*, *rps19*, *^,a^*rpl2*, *rpl14*, * *rpl16*, *rpl20*, *rpl22*, ^a^ *rpl23*, *rpl32*, *rpl33*, *rpl36*
	translation initiation factor	*infA*
RNA genes	ribosomal RNA	^a^*rrn5*, ^a^ *rrn4.5*, ^a^ *rrn16*, ^a^ *rrn23*
	transfer RNA	*^,a^ *trnA-UGC*, *^#^ trnA-ACG*, *trnL-UAG*, *^a^trnA-GUU*, *^,*a*^*trnI-GAU*, *^a^ trnV-(GAC)*, *^a^ trnL-CAA*, ^a^ *trnH-CAU*, *trnP-UGG*, *trnT-CCA*, *trnM-CAU*, * *trnV-UAC*, *trnP-GAA*, * *trnL-UAA*, *trnT-UGU*, *trnS-GGA*, *trnfM-CAU*, *trnG-GCC*, *trnS-UGA*, *trnT-GGU*, *trnG-UUC*, *trnT-GUA*, *trnA-GUC*, *trnC-GCA*, *trnA-UCU*, * *trnG-UCC*, *trnS-GCU*, *trnG-UUG*, * *trnL -UUU*, *trnH-GUG*, *trnA-GUU*
Other genes	RNA processing	*matK*
	carbon metabolism	*cemA*
	fatty acid synthesis	*accD*
	proteolysis	** *clpP*
Genes of unknown function	conserved reading frames	^a^ *ycf1*, ^a^ *ycf2*, *ycf4*, ^a^ *ycf15*

* gene with one intron, ** gene with two introns, ^a^ gene with two copies.

## Supplementary Materials

The following supplementary materials can be found at www.mdpi.com/2073-4425/8/3/103/s1, Table S1: Primers used for gap closure and *rpoC2* gene verification; Table S2: Repetitive sequence statistics; Table S3: Ka/Ks ratio between pairwise of species protein coding sequences in six *Rehmannia* species; Figure S1: Alignment of *rpoC2* fragments from all six species of *Rehmannia*. Large indels were detected in the *rpoC2* gene, which cause the gene size vary from 2916 bp to 4185 bp among the six species; Figure S2: The Mauve alignment between plastomes of *Arabidopsis thaliana* and *Rehmannia* species (A) and between those of *A. thaliana* and core Lamiales taxa (B).
