# Supplementary material for: The Complete Chloroplast Genome Sequences of Six Rehmannia Species
Source: Genes (Basel). 2017 Mar 15;8(3):103. doi: 10.3390/genes8030103 (PMC5368707; doi:10.3390/genes8030103)
Supplement: Supplementary file 1 [file genes-08-00103-s001.zip › supplement files/Table S1.docx]

**Table S1. Primers used for gap closure and *rpoC2* gene verification**.

| **Primer** | **Forward sequence (5' to 3')** | **Reverse sequence (5' to 3')** | **Production size (bp)** |
| --- | --- | --- | --- |
| 1 | ATTCCATTTCCCCTGACTCC | GCATCTTCTCCTTGGCAAAG | 500 |
| 2 | CTATTCCAAATCACGCGAGC | CGAATAAAAGAAGGCGCCAT | 450 |
| 3 | ATGCTCTAACCTCTGAGC | TTTGTTTTGAGATTAGGATC | 600 |
| 4 | TACTGTAGATTGGCCGTAGA | AAGGTGTTGGTTTCAGAAGA | 540 |
| 5 | CGTTCTCTTTCAAGCCTTTC | TGCAACTTGACAATTTAAAAGAG | 830 |
| 6 | TCGAACGCACTTCTAACACC | CCGGATCTGTTGCACGAAAG | 1021 |
| 7 | CACGGGCTTCTTGACTAG | CAATTGATTCAAGAACGCGA | 750 |

Primer pairs 1-3 were used to fill gaps in the assembly of *Rehmannia glutinosa*. Primer pairs 4-7 were used in sequence verification of *rpoC2* gene in *Rehmannia* species.

Ambiguous (N) bases of *Rehmannia* *glutinosa* cp genomes were corrected by PCR amplification and Sanger sequencing. A high variable insertion/deletion (indel) region was found in *rpoC2* gene. To validate the sequence of *rpoC2* gene, 6 *rpoC2* sequences of *Rehmannia* were aligned with program MEGA 6.0 and primers were designed in the conserved region using Primer3. The PCR reaction was performed in a thermocycler (Veriti, Applied Biosystems, CA, USA) using the following cycling parameters: 5 min for 94°C; 35 cycles of 94°C (30 s), 55°C (30 s), 72°C (1 min); and final extension at 72°C (10 min). PCR products were sequenced by Bionics Co. (Seoul, South Korea).
