# Supplementary material for: The Complete Chloroplast Genome Sequences of Six Rehmannia Species
Source: Genes (Basel). 2017 Mar 15;8(3):103. doi: 10.3390/genes8030103 (PMC5368707; doi:10.3390/genes8030103)
Supplement: Supplementary file 1 [file genes-08-00103-s001.zip › supplement files/Table S3.docx]

**Table S3. Ka/KS between six species of *Rehmannia* protein coding sequences.**

|  | *Rg-Rc* | *Rg-Re* | *Rg-Rh* | *Rc-Rh* | *Re-Rh* | *Rg-Rp* | *Rc-Rp* | *Re-Rp* | *Rh-Rp* | *Rg-Rs* | *Rc-Rs* | *Re-Rs* | *Rp-Rs* |
| --- | --- | --- | --- | --- | --- | --- | --- | --- | --- | --- | --- | --- | --- |
| *accD* | 0.236 | 0.236 | **1.182** | - | - | 0.237 | - | - | - | **1.182** | - | - | - |
| *ccsA* | 0.248 | 0.248 | 0.000 | 0.124 | 0.124 | 0.000 | - | - | 0.000 | 0.000 | 0.124 | 0.124 | 0.000 |
| *cemA* | - | - | - | - | - | 0.470 | 0.234 | 0.234 | 0.234 | - | - | - | 0.234 |
| *clpP* | 0.000 | 0.000 | - | 0.000 | 0.000 | - | 0.284 | 0.284 | - | - | 0.000 | 0.000 | - |
| *matK* | 0.514 | 0.514 | 0.768 | - | - | 0.514 | - | - | - | 0.768 | - | - | - |
| *ndhA* | 0.294 | 0.294 | 0.882 | 0.293 | 0.293 | 0.294 | - | - | 0.293 | 0.882 | 0.293 | 0.293 | 0.293 |
| *ndhC* | - | - | 0.000 | 0.000 | 0.000 | - | - | - | 0.253 | 0.000 | 0.000 | 0.000 | 0.253 |
| *ndhD* | - | - | 0.000 | 0.554 | 0.554 | 0.276 | 0.831 | 0.831 | 0.138 | 0.000 | 0.554 | 0.554 | 0.138 |
| *ndhF* | 0.657 | 0.657 | 0.458 | 0.262 | 0.262 | 0.525 | 0.263 | 0.263 | 0.262 | 0.458 | 0.262 | 0.262 | 0.262 |
| *ndhK* | - | - | 0.000 | 0.284 | 0.284 | - | - | - | 0.000 | 0.000 | 0.284 | 0.284 | 0.000 |
| *petA* | 0.000 | 0.000 | 0.000 | 0.000 | 0.000 | 0.281 | - | - | 0.281 | 0.000 | 0.000 | 0.000 | 0.281 |
| *psaA* | 0.000 | 0.000 | 0.071 | 0.040 | 0.040 | - | 0.094 | 0.094 | 0.141 | 0.071 | 0.040 | 0.040 | 0.141 |
| *psaB* | 0.136 | 0.136 | 0.090 | 0.000 | 0.000 | 0.180 | 0.272 | 0.272 | 0.136 | 0.090 | 0.000 | 0.000 | 0.136 |
| *psbK* | - | - | - | - | - | 0.000 | 0.000 | 0.000 | 0.511 | - | - | - | 0.511 |
| *rbcL* | - | - | 0.290 | 0.290 | 0.290 | 0.872 | 0.872 | 0.872 | 0.481 | 0.290 | 0.290 | 0.290 | 0.481 |
| *rpl14* | - | - | 0.280 | 0.280 | 0.280 | - | - | - | 0.280 | 0.280 | 0.280 | 0.280 | 0.280 |
| *rpl22* | 0.259 | 0.259 | 0.259 | - | - | 0.521 | - | - | - | 0.259 | - | - | - |
| *rpoA* | **1.242** | **1.242** | - | 0.495 | 0.495 | 1.237 | 0.246 | 0.246 | 0.494 | - | 0.495 | 0.495 | 0.494 |
| *rpoB* | 0.553 | 0.553 | 0.276 | 0.553 | 0.553 | 0.207 | 0.276 | 0.276 | 0.207 | 0.276 | 0.553 | 0.553 | 0.207 |
| *rpoC1* | 0.272 | 0.272 | 0.182 | 0.548 | 0.548 | 0.091 | 0.274 | 0.274 | 0.137 | 0.182 | 0.548 | 0.548 | 0.137 |
| *rpoC2* | 0.273 | 0.382 | 0.429 | 0.818 | 1.092 | 0.492 | - | - | **1.364** | 0.429 | 0.818 | **1.092** | **1.364** |
| *rps3* | - | - | 0.241 | 0.240 | 0.240 | - | - | 0.000 | 0.241 | 0.240 | 0.240 | 0.240 | 0.000 |
| *rps15* | 0.000 | 0.000 | 0.261 | 0.261 | 0.261 | 0.261 | 0.261 | - | 0.261 | 0.261 | 0.261 | 0.261 | - |
| *psbB* | - | - | 0.289 | 0.289 | 0.289 | 0.000 | 0.000 | 0.000 | 0.144 | 0.289 | 0.289 | 0.289 | 0.144 |
| *psbD* | 0.000 | 0.000 | 0.289 | 0.143 | 0.143 | - | 0.000 | 0.000 | 0.289 | 0.289 | 0.143 | 0.143 | 0.289 |

*Rg*, *R. glutinosa*; *Rc*, *R. chingii*; *Rs*, *R. solanifolia*; *Rh*, *R. henryi*; *Re*, *R. elata*; *Rp*. *R. piasezkii*.
