# Supplementary figures and images for: The Complete Chloroplast Genome Sequences of Six Rehmannia Species
Source: Genes (Basel). 2017 Mar 15;8(3):103. doi: 10.3390/genes8030103 (PMC5368707; doi:10.3390/genes8030103)

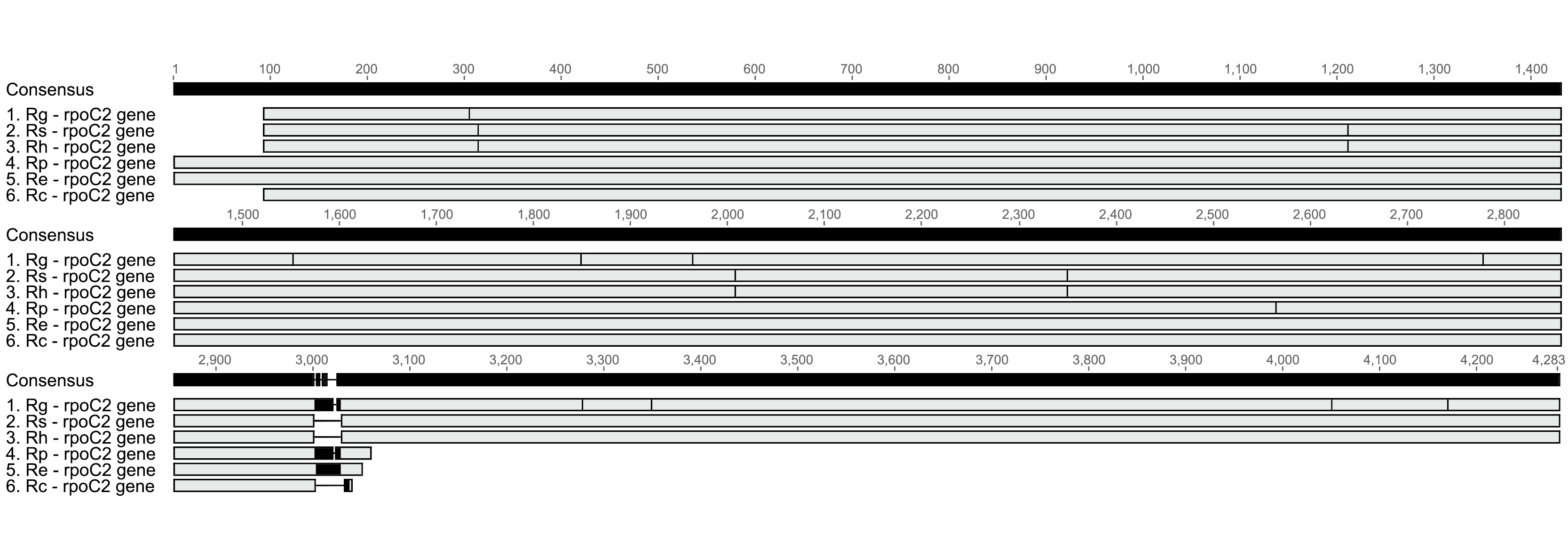

Supplement: Supplementary file 1 [file genes-08-00103-s001.zip › supplement files/Fig S1.tif]

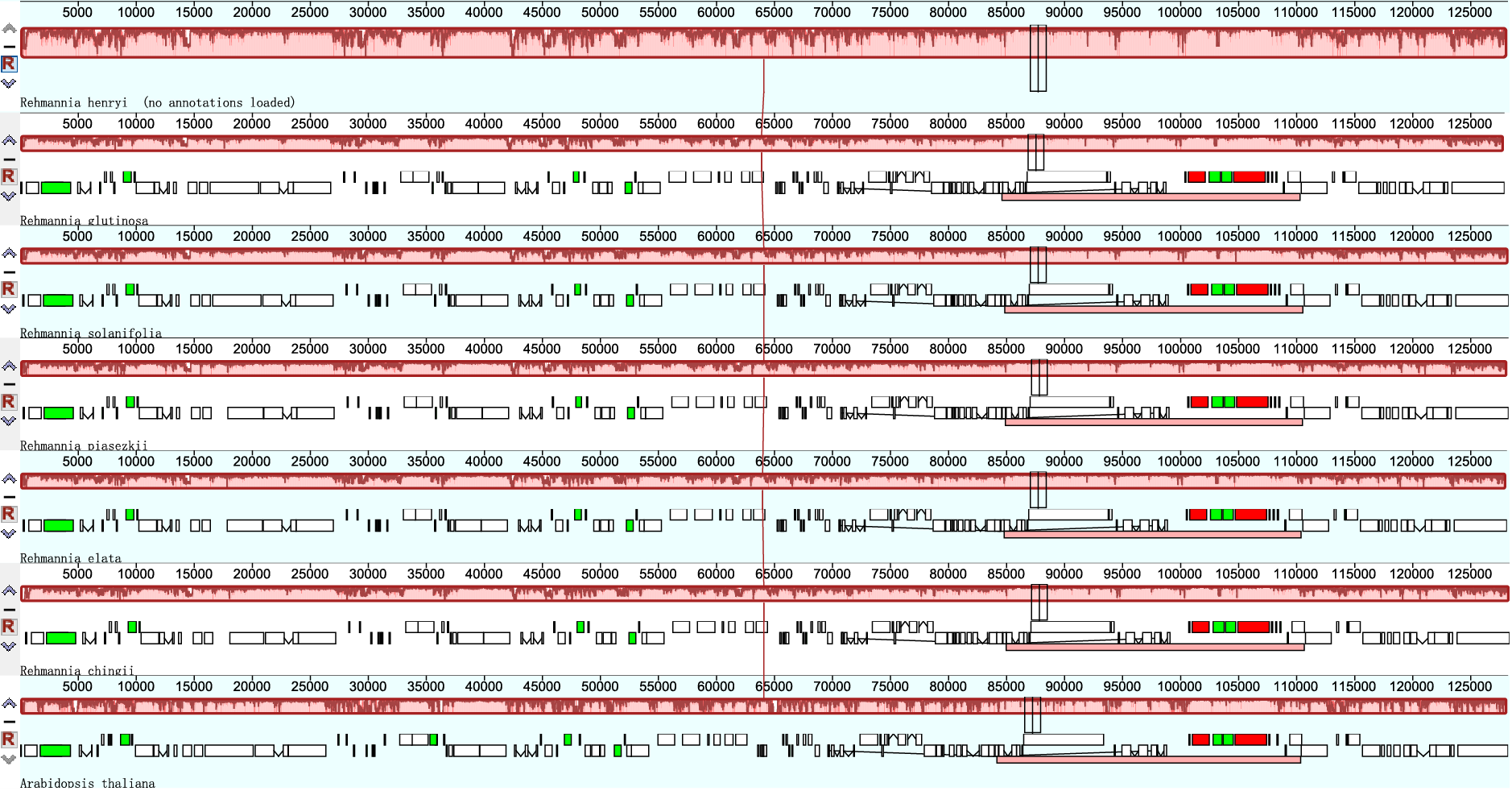

Supplement: Supplementary file 1 [file genes-08-00103-s001.zip › supplement files/Fig S2/Figure S2 A.tif]

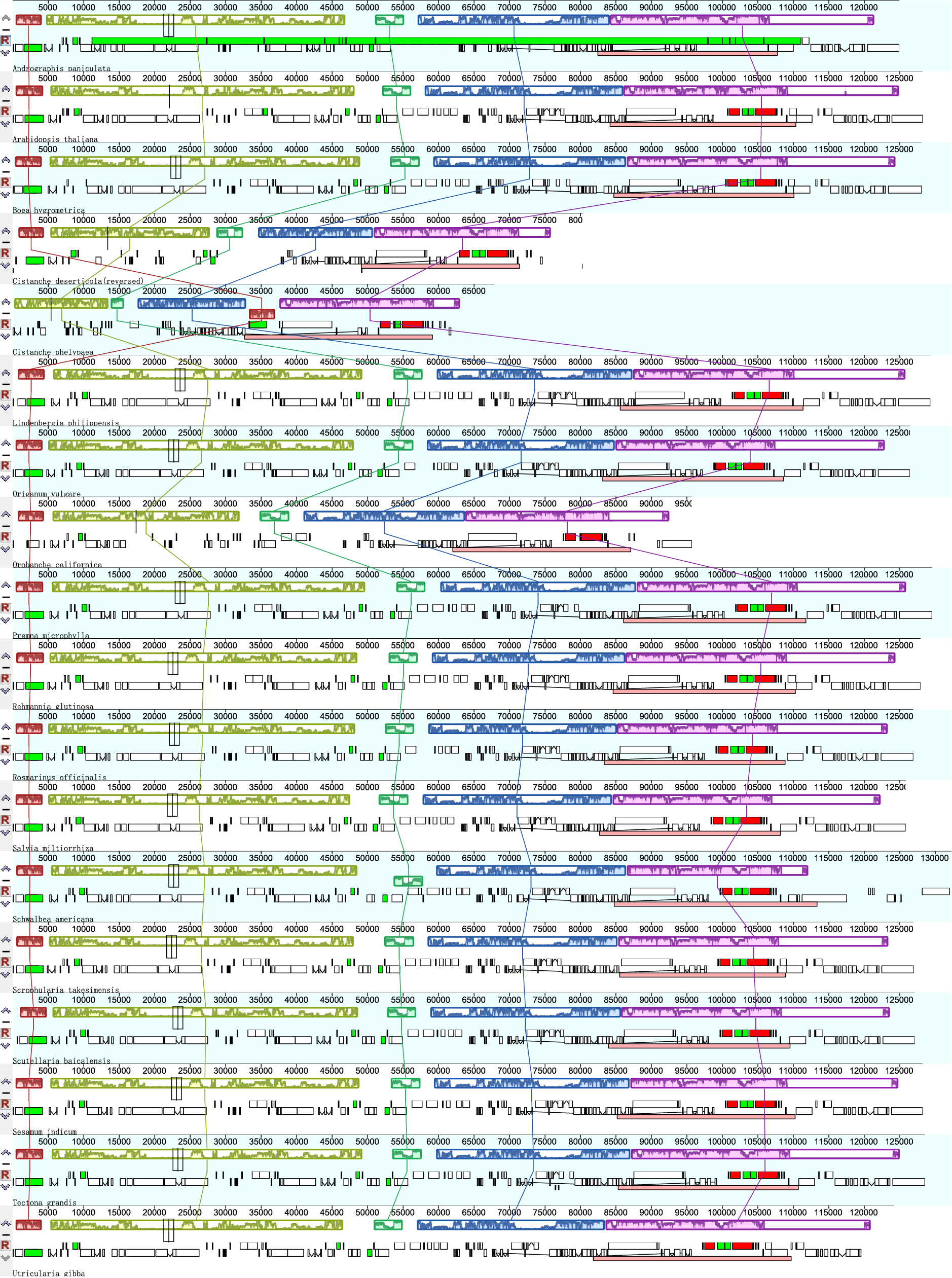

Supplement: Supplementary file 1 [file genes-08-00103-s001.zip › supplement files/Fig S2/Figure S2 B.tif]
